# Comparing Effects of Lake- and Watershed-Scale Influences on Communities of Aquatic Invertebrates in Shallow Lakes

**DOI:** 10.1371/journal.pone.0044644

**Published:** 2012-09-06

**Authors:** Mark A. Hanson, Brian R. Herwig, Kyle D. Zimmer, John Fieberg, Sean R. Vaughn, Robert G. Wright, Jerry A. Younk

**Affiliations:** 1 Wetland Wildlife Populations and Research Group, Minnesota Department of Natural Resources, Bemidji, Minnesota, United States of America; 2 Division of Fish and Wildlife – Fisheries Research, Minnesota Department of Natural Resources, Bemidji, Minnesota, United States of America; 3 Department of Biology, University of St. Thomas, St. Paul, Minnesota, United States of America; 4 Biometrics Unit, Minnesota Department of Natural Resources, Forest Lake, Minnesota, United States of America; 5 Division of Waters, Minnesota Department of Natural Resources, Cambridge, Minnesota, United States of America; 6 Wildlife GIS Unit, Minnesota Department of Natural Resources, Forest Lake, Minnesota, United States of America; Federal University of Rio de Janeiro, Brazil

## Abstract

Constraints on lake communities are complex and are usually studied by using limited combinations of variables derived from measurements within or adjacent to study waters. While informative, results often provide limited insight about magnitude of simultaneous influences operating at multiple scales, such as lake- vs. watershed-scale. To formulate comparisons of such contrasting influences, we explored factors controlling the abundance of predominant aquatic invertebrates in 75 shallow lakes in western Minnesota, USA. Using robust regression techniques, we modeled relative abundance of Amphipoda, small and large cladocera, Corixidae, aquatic Diptera, and an aggregate taxon that combined Ephemeroptera-Trichoptera-Odonata (ETO) in response to lake- and watershed-scale characteristics. Predictor variables included fish and submerged plant abundance, linear distance to the nearest wetland or lake, watershed size, and proportion of the watershed in agricultural production. Among-lake variability in invertebrate abundance was more often explained by lake-scale predictors than by variables based on watershed characteristics. For example, we identified significant associations between fish presence and community type and abundance of small and large cladocera, Amphipoda, Diptera, and ETO. Abundance of Amphipoda, Diptera, and Corixidae were also positively correlated with submerged plant abundance. We observed no associations between lake-watershed variables and abundance of our invertebrate taxa. Broadly, our results seem to indicate preeminence of lake-level influences on aquatic invertebrates in shallow lakes, but historical land-use legacies may mask important relationships.

## Introduction

Inland freshwaters are increasingly recognized for the ecosystem services they provide, yet, the integrity of these systems is threatened at a global scale [1], [2]. Watershed features are thought to influence composition and well-being of lake communities, but these relationships are poorly documented [3]. More research has explored relationships among lake water quality, watershed nutrient transport, and other lake watershed variables such as land use patterns [4]–[6]. Despite strong interests in conservation and restoration, ecologists have rarely assessed linkages between watershed attributes and aquatic invertebrate communities residing in freshwater lakes [3]. Almost certainly, future conservation strategies will seek to link aquatic communities to pervasive stressors such as climate change, alteration of upland cover types and composition, agriculture, and even modification of lake-watershed configurations. Given roles of aquatic invertebrates in nutrient dynamics and aquatic food webs, understanding of patterns between these communities and landscape mosaics in lake watersheds is a key step toward conservation of freshwater lakes [2], [3], [7].

Aquatic invertebrates are key biological components of freshwater ecosystems worldwide [8], [9] and are among the most abundant fauna in wetlands and shallow lakes where they comprise a major share of the biodiversity [10]. Aquatic insects and crustaceans play roles in cycling nutrients and organic matter, and comprise critical products of secondary production in aquatic and terrestrial food webs [8], [11]. Invertebrates graze on periphyton, thus helping to sustain submerged macrophyte communities in nutrient-rich shallow lakes [12]–[14]. Important lake features may depend on a small number of key invertebrate taxa and these organisms may be sensitive to changes in lake characteristics. For example, by grazing on phytoplankton, large-bodied herbivorous zooplankton influence seasonal patterns of water clarity, at least in lakes with low to moderate levels of phosphorus. Increased fish density often reduces zooplankton density and herbivory, favoring algal blooms and reducing likelihood of clear-water regimes in shallow lakes [15]–[19].

Shallow lakes are widespread throughout the Prairie Pothole Region (PPR) of North America, an area encompassing approximately 700,000 km^2^. Minnesota has thousands of PPR lakes characterized by mean depth ≤4.5 m and surface area >16.2 hectares (Nicole Hansel-Welch, Minnesota Department of Natural Resources, personal communication, 2012). These lakes are strongly influenced by hydrogeomorphic setting and climate, especially precipitation [20], and fish populations are known to exert major influences on resident communities [21]–[23]. The lakes represent an international resource, providing critical waterfowl habitat and ecological benefits within the central U.S and Canada. Here and elsewhere, water quality has declined dramatically and this trend continues today [24]–[26]. Shallow lakes offer unique challenges for lake managers [23], [27], due to pressure from anthropogenic development and other stressors, and because land-use legacies may limit success of lake rehabilitation efforts [28].

Here, we describe results of a 2-year study of factors influencing abundance of aquatic invertebrate taxa in 75 shallow lakes within 2 ecoregions of Minnesota. Our main objective was to compare the relative importance of watershed-scale variables and variables measured at a within-lake scale. We used robust regression techniques to quantify associations between invertebrate abundance and lake watershed size (area), extent of geographic isolation, proportion of agriculture within watersheds, fish abundance and community composition, and submerged macrophytes. We also explored predicted and residual variance from regression models to help evaluate alternative mechanisms for observed patterns.

## Materials and Methods

### Study Area and Study Sites

**Figure 1:**
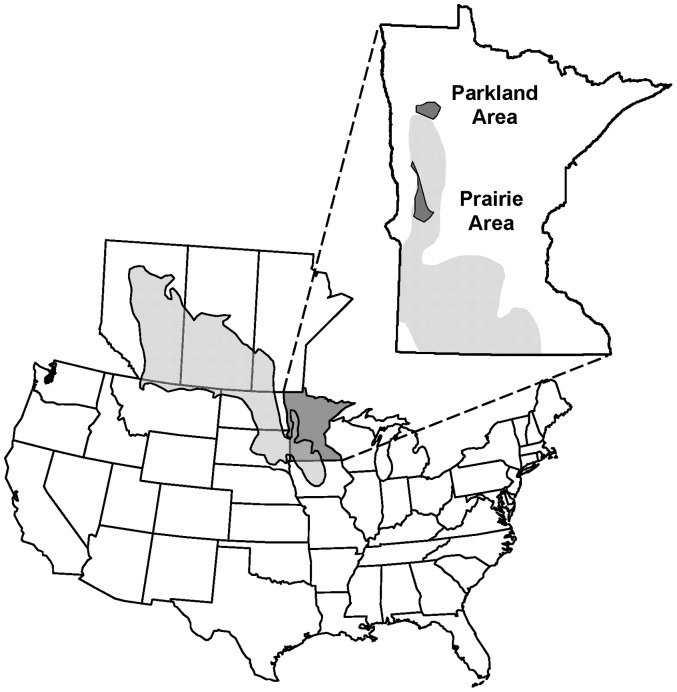
Location of shallow study areas. Map showing locations of study lakes within parkland (north, 95°25′N 45°40′W) and prairie (south, 95°60′N 47°30′W) study regions in Minnesota, USA. Shaded area indicates extent of Prairie Pothole Region.

We gathered data from shallow lakes along the eastern margin of the PPR of North America within two contrasting ecoregions in western Minnesota, USA (Fig. 1). Our northern lakes were set within a prairie-parkland transition (hereafter parkland); a southern study area was located within a prairie zone (hereafter prairie) [29]. Lakes in these ecoregions lie within thick glacial till, and precipitation exceeds evaporation in most years [21]. Parkland and prairie study regions differed in geomorphic features, climate, upland vegetation patterns, and lake nutrient levels, with much higher mean nitrogen and phosphorus in prairie lakes [30]. Parkland and prairie study regions encompassed approximately 1,435 km^2^ and 1,292 km^2^, respectively. Study lakes occurred within lands owned by the United States Fish and Wildlife Service (USFWS), the Minnesota Department of Natural Resources, and private individuals. Necessary permits for sampling on federal lands were obtained directly from the USFWS. Permission to access lakes on private lands was obtained from individual landowners.

We identified all Type 4 and 5 lakes [31] from 2–50 ha within each of the 2 study regions using a National Wetlands Inventory GIS database. We randomly selected 75 lakes based on size of open water surface area (small, medium, large, range 2–50 ha), proximity to nearest permanent stream, wetland, or lake (short, medium, long, range 0 to 1.8 km), and proportion agriculture (identified on aerial photographs as areas supporting hay, small grains or row crops such as corn and soybeans) within lake watersheds. Study lakes were typical of those common throughout these ecological regions and ranged in surface area (including fringe of emergent vegetation) from 3.6–93.1 ha in the parkland (mean = 19.6 ha) and from 1.6–47.7 ha in the prairie (mean = 15.6 ha). Maximum depths ranged from 0.6–7.5 m in parkland (mean = 2.7 m) and from 0.5–4.6 m in the prairie (mean = 1.8 m) lakes. Mean total phosphorus (TP) concentrations differed markedly between the study areas, averaging 47.6 µg•L^−1^ (SE = 8.3) and 179.2 µg•L^−1^ (SE = 24.2) in the parkland and prairie study regions, respectively.

### Data Collection

#### Within-lake characteristics

Fish presence and community composition were determined from surveys in July each year using a combination of gears deployed overnight. During 2005–06, 3 mini-fyke nets (6.5 mm bar mesh with 4 hoops, 1 throat, 7.62 m lead, and a 0.69 m×0.99 m rectangular frame opening into the trap) were set in the littoral zone of each lake. One experimental gill net (61-m multifilament net with 19, 25, 32, 38, and 51-mm bar meshes) was set along the deepest depth contour available in lakes <2 m deep or along a 2-m contour in sites with sufficient depth. This approach has been shown to be effective in sampling fish assemblages in small lakes from other regions [32]–[35] and enabled us to capture fish of different sizes, species, and from all major trophic guilds (i.e., planktivores, benthivores, piscivores). Total biomass of each species collected was determined for each type of gear and summed to produce biomass estimates of relative abundance (CPUE) for each lake. We ranked fish communities in each lake by assigning a class score from 0–3 to depict the trophic guilds present (0 = fishless, 1 = planktivores only (P), 2 = planktivores+benthivores (PB), and 3 = planktivores+benthivores+ piscivores (PBP)). Species were assigned to trophic guilds based on published diet data from lake systems [36], [37].

We assessed community-level biomass of submerged macrophytes using raking techniques of [38]. Submerged macrophytes, including macroalgae (*Chara* spp.), were sampled in each lake during July or early August at 20 stations equidistant along 4 transects. One rake cast was made at each station using a weighted plant rake and the total plant biomass (wet weight of all species combined) was determined. Values were summed to produce a lake-wide plant mass score.

#### Watershed-scale spatial data

Lake surface extent and internal open water areas were calculated from digitization of these features as interpreted from aerial photographs (2003 Farm Service Agency color digital orthophoto quadrangles [DOQs]) using GIS software (ArcView 3.3 and ArcGIS 9.2 [39]). We defined lake watersheds as lands (including all water features) having potential to contribute excess water from the surface water system to the outlet of each of our study sites. Lake watersheds were determined using height of land delineation techniques and on-screen heads-up digitizing procedures in ArcGIS 9.2. Data sources used to delineate these lake watersheds included USGS 1∶24,000 digital raster graphic “topo” maps (used as a base layer), digital aerial photography, hydrologically-corrected digital elevation models derived via ANUDEM software [40] and existing stream, lake, dam, National Wetland Inventory, and Minnesota Department of Transportation culvert GIS data bases [41]. Using National Wetland Inventory GIS data bases and tools within ArcGIS, we measured distance from each study lake to the nearest type 4 or 5 wetland [31] or the nearest lake (surface area >100 acres). Finally, using 2003 DOQ’s within ArcGIS, we used on-screen digitizing procedures to delineate upland cover types within study lake watersheds and assigned resulting polygons to agriculture (row crops, hay lands, etc.) and a wide range of other cover types.

#### Sampling aquatic invertebrates

We collected aquatic invertebrates in all lakes during July each year (2005–06) using integrated column samplers (CS) and activity traps (AT). Zooplankton was sampled by collecting 2 replicate vertical CSs [42] at each of 5 or 6 locations in each lake. Relative abundance of free-swimming macroinvertebrates was assessed concurrently using submerged ATs [43] in each lake for 24-hours. ATs were deployed at 5 or 6 locations along the interface of open water and emergent macrophytes. Estimates of relative invertebrate abundance were developed for each lake by combining data from ATs and CSs to form a single annual value.

### Data Organization and Statistical Analyses

We chose 6 predictor variables *a priori* to represent potential in-lake (3) and watershed-scale influences (3) based on their documented or presumed relationships to aquatic ecosystems. Predictor variables were: 1) fish community class (identified as 0, P, PB, PBP; [19], [44]–[47], 2) mass of planktivorous+ benthivorous fishes (index to abundance) [21], [48]–[53], 3) relative abundance of submerged macrophytes [13], [14], [53]–[55], 4) lake watershed size (surface area; [4], [5], [56], [57], 5) spatial isolation (straight-line distance to nearest wetland or lake) [32], [58]–[62], and 6) proportion of agriculture in the study lake watershed [5], [6], [63], [64], [65]. Fish community class was represented using a set of 4 dummy regression parameters to reflect differences between classes (0 = fishless, 1 = P, 2 = PB, 3 = PBP); class = 0 served as the baseline (i.e., reference). We included region as a fixed-effect variable in our regression models to account for differences in watershed nutrient levels and upland cover types between our 2 study regions.

We focused on 6 aggregate taxa as invertebrate response variables. Use of aggregate variables is often necessary for univariate models because these methods perform poorly with infrequently sampled taxa. Main groups of invertebrates included: small cladocerans (*Bosmina, Chydorus, Eubosmina*), large cladocerans (*Daphnia, Ceriodaphnia, Simocephalis, Scaphelaberis*), amphipods (*Gammarus*, *Hyalella*), Diptera (Ceratopogonidae, Chaoboridae, Chironomidae, Tipulidae), Corixidae, and ETO (Ephemeroptera+Trichoptera+ Odonata). Our taxon ETO was similar to variables used previously for development of invertebrate IBI metrics for wetlands in Minnesota and elsewhere [66]–[68].

Before fitting any models to the data, we used variance inflation factors to look for potential multicollinearity [69]. In addition, we noted that the response distributions were highly skewed, and often contained large outliers. We square-root transformed the responses, which limited but did not completely eliminate skewness or potential influence of outlying observations.

We used a robust linear model fitting technique [70] implemented via the lmRob function in the robust library [71] of Program R [71] to assess the importance of each of the 6 predictor variables. We fit separate models to each square root-transformed response variable, including the same set of predictors in each case. We examined residuals plotted against each predictor variable, along with smooth trend lines using the lowess function in Program R [72] to look for patterns that might suggest a need to model non-linear effects. To evaluate statistical significance of parameter estimates, we used a non-parametric bootstrap in which we re-sampled lakes to account for potential within-lake correlations, refitting each invertebrate response model to each of 10,000 bootstrap data sets [73]. At least 1 fishless lake had to be present to allow all parameters to be identifiable. We rejected all data sets (<0.6%) that did not include a fishless lake and continued sampling until a total of 10,000 bootstrap data sets had been achieved.

We summarized model results (comparing predictor influences on invertebrates) by plotting percentile-based 95% bootstrap confidence intervals for regression parameters (slope estimates). Strong effects of fish on invertebrate communities have been shown for regional shallow lakes [46], [52], [53]. Thus, we also explored abundance patterns for each of our 6 invertebrate taxa using side-by-side boxplots (depicting invertebrate abundance across our fish community classes). Unfortunately, our random selection of study sites produced only 5 fishless lakes. Therefore, we also included (in the boxplots only) supplemental data from 8 fishless lakes similarly studied within our prairie area. Supplemental lakes were sampled during 1999 using comparable methods [18].

## Results

**Figure 2:**
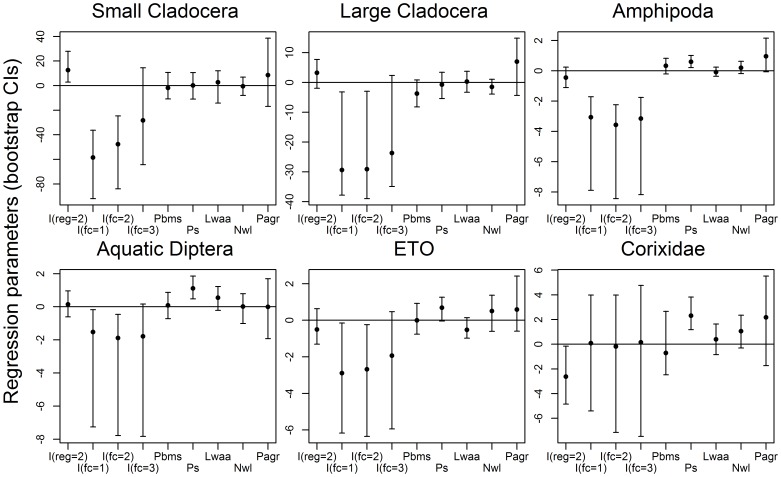
Confidence intervals for parameter estimates from regression models of invertebrate variables. Percentile-based 95% bootstrap confidence intervals for regression parameters associated with each of six invertebrate response variables: small cladocerans, large cladocerans, Amphipoda, Diptera, ETO = Emphemeroptera+Trichoptera+Odonata, Corixidae. For each response, the following predictors were included in the model: I(reg = 2) = an indicator variable equal to 1 if the lake is in the Parkland region and 0 if from the Prairie region [reference level]), I(fc = i) = an indicator variable equal to 1 if fish class = i and 0 otherwise (for i = 1, 2, 3), Pbms = planktivore+benthivore biomass (log transformed), Ps = submerged plant score, Lwaa = surface area of upstream lake watershed, Nwl = distance to the nearest permanent wetland or lake, Pagr = proportion agriculture within the upstream lake watershed. See Methods for additional details.

Lake-scale variables showed stronger influences on aquatic invertebrate abundance than did watershed-scale features. Fish community classification and submerged plant predictor variables were most often related to the abundance of aquatic invertebrates we surveyed. Contrary to our expectations, lake watershed size, extent of isolation, and proportion of watershed in agriculture showed no significant relationships to abundance of our invertebrate groups (in all cases, confidence intervals overlapped 0). Abundance of most invertebrate taxa was similar between study regions (although small cladocera and Corixidae were more abundant in Parkland and Prairie regions, respectively; Fig. 2). Models for submerged plant mass had positive parameter estimates for Amphipoda, aquatic Diptera, and Corixidae, indicating that these taxa were more abundant in lakes with robust plant communities (Fig. 2).

**Figure 3:**
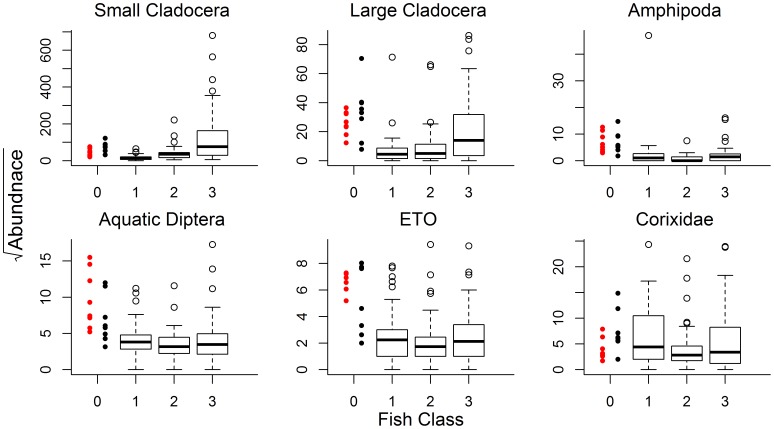
Aquatic invertebrate abundance among lakes with different fish communities. Box plots depicting relative abundance patterns for each of 6 invertebrate response variables (ETO = Emphemeroptera+Trichoptera+Odonata). Fish class indicates fish absence (value = 0) or whether community contained only planktivores (value = 1), planktivores +benthivores (value = 2), or planktivores+benthivores+piscivores (value = 3). Abundance estimates for fishless lakes include supplemental data (values in red = 1999; black = current study) and are included to allow comparisons with fishless lakes. See text for more explanation.

Regression coefficients for fish presence were largely negative, regardless of fish community class (Fig. 2), suggesting invertebrate abundances tended to be highest in fishless lakes. These conclusions were also supported by plots of the raw distribution of invertebrate abundances among fish classes (Fig. 3), including those from fishless lakes sampled in 1999 during a previous study in the same prairie area (Fig. 3; [18]).

Fish populations were widespread throughout shallow lakes in our 2 study regions and were present in all but 5 (7%) of our primary study lakes. Fathead minnow, brook stickleback, and black bullhead were most ubiquitous, all occurring in ≥40% of our lakes. Most common planktivores were fathead minnow *Pimephales promelas*, brook stickleback *Culea inconstans*, central mudminnow *Umbra limi*, yellow perch *Perca flavescens*, and northern redbelly dace *Phoxinus eos*. Predominant benthivores were black bullhead *Ameiurus melas*, white sucker *Catostomas commersoni*, and common carp *Cyprinus carpio*. Northern pike *Esox lucius*, largemouth bass *Micropterus salmoides*, and walleye *Sander vitreus* were the only piscivores.

**Figure 4:**
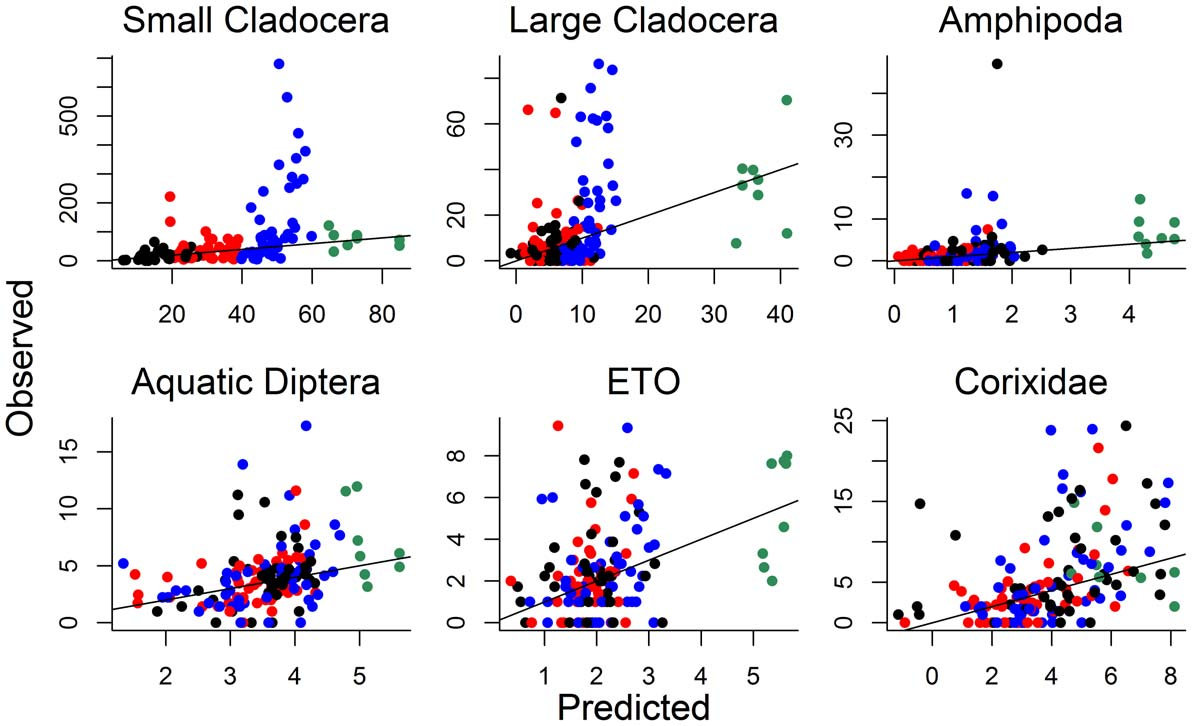
Comparisons of observed and predicted values from regression models. Observed vs. predicted values from fitted (robust regression) models, along with a 1∶1 line. Colors depict different fish classifications: green = fishless, black = planktivores, red = planktivores+benthivores, blue = planktivores+benthivores+piscivores.

**Figure 5:**
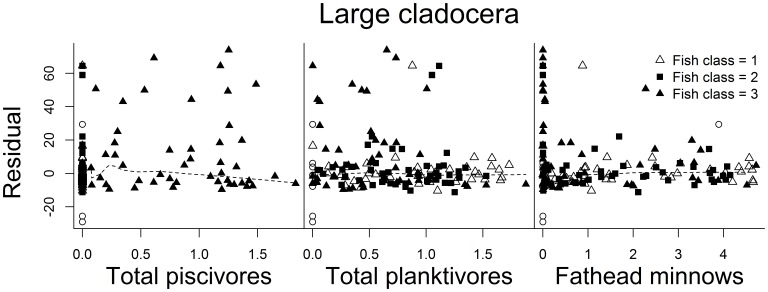
Relationships between large cladoceran model predictions and fish communities in study lakes. Residual values from the large cladoceran model versus relative abundance (mass) of total piscivores, total planktivores, and fathead minnows. Lines depict smooth trends estimated using the “lowess” function in program R. Symbol depicts fish class or fish feeding guild present in lakes (0 =  fishless, 1 = planktivores only, 2 = planktivores and benthivores, 3 = planktivores, benthivores, and piscivores).

Finally, we plotted observed versus model-based predicted values for each invertebrate taxon, using color to indicate fish class (0–3) (Fig. 4). We also plotted residual values from the large cladoceran model against abundance of piscivores, planktivores, and fathead minnows (Fig. 5), because strong trophic cascades of fish on *Daphnia* and other large-bodied zooplankton have been shown in regional shallow lakes [17], [19] and we expected similar effects here. Large residuals were evident in all models (except perhaps for amphipods), but more so for small and large cladocera (Fig. 4). However, residual values showed little relationship to relative abundance of piscivores, planktivores, or fathead minnows in our study lakes (Fig. 5).

## Discussion

Our study is one of the first to compare roles of in-lake influences along with watershed-scale factors as determinants of broad patterns of invertebrate abundance in shallow lakes. In general, we found that in-lake measurements more often explained among-lake variability in invertebrate abundance than did watershed-scale factors. For example, we identified significant associations between fish presence and community type and abundance of small and large cladocera, Amphipoda, Diptera, and ETO. Abundance of Amphipoda, Diptera, and Corixidae were also positively correlated with submerged plant abundance. In contrast, we observed no consistent associations between lake-watershed variables and abundance of our invertebrate taxa.

Ecological characteristics of north-temperate shallow lakes have been extensively studied during the last 2 decades, with much research focused on identifying causes of rapid shifts toward turbid regimes, the latter being universally considered to indicate poor water quality and habitat conditions [23], [28], [52], [74], [75]. Along with the realization that shallow lakes are often subject to anthropogenic eutrophication and runoff waters high in agricultural chemicals [4]–[6], this has stimulated interest in reversing turbid shifts, improving water transparency, and restoring natural communities of rooted aquatic plants, birds, fish, amphibians, and aquatic invertebrates. Most restoration efforts have focused on limiting point source nutrient inputs, removing sediments, or reducing planktivorous and benthivorous fishes to increase zooplankton grazing pressure on phytoplankton and to reduce resuspension of sediments and nutrients [74]. As predicted by theoretical models of alternative stable states with hysteresis, restoring shallow lakes has proven difficult because turbid lakes show strong resilience and resist transition back to clear regimes [23], [74].

To date, the most successful lake restoration efforts (at least over short time periods) have targeted in-lake aspects, especially sharp reductions in planktivore/benthivore removals [74], [76]. There is some evidence, especially from European lakes, that nutrient load reduction also has potential to overcome resilience of turbid regimes and favor shifts to clear-water conditions [76] and that nutrient reduction may be a requirement for long-term reestablishment of clear regimes [28], [74]. However, studies of shallow lake community responses to watershed features are rare (although zooplankton richness and composition have been shown to be influenced by extent of vegetated uplands and perhaps primary productivity of watersheds surrounding small lakes [3], [77]).

We expected our models to reflect changing abundance of invertebrates along gradients of increasing watershed size, isolation, and perhaps extent of agriculture. Surprisingly, invertebrate abundances showed no negative relationships to those variables, although positive abundance trends were observed for large cladocera, Amphipoda, and Corixidae with increasing agriculture (yet not significant at α  = 0.05). There are at least 2 reasons why we believe our models failed to identify relationships between abundance of important invertebrate taxa and watershed-scale predictors (isolation, lake-watershed area, and the proportion of upstream agriculture). First, shallow lakes in our study region have been shown to conform to predictions of alternative stable regime models [52] and show strong evidence of biological-based resilience (such as robust planktivore and benthivore populations [76]) thus promoting turbid regime lake characteristics, including reduced invertebrate populations. Second, and contributing to nutrient-based resiliency, historical land-use legacies have profoundly altered ecological characteristics of regional shallow lakes such that present ecological characteristics of these turbid lakes are probably uncoupled from contemporary features of lake watersheds. In portions of central North America, >95% of original grasslands have been converted to agriculture [78] and thousands of surface waters have been eliminated entirely by surface and subsurface drainage [21], [65]. Agriculture contributes large quantities of nutrients and modifies nutrient ratios in lakes within affected watersheds [5], [6], [26], [63], [79] and elevated nutrients may increase lake trophic status and favor turbid regimes [53]. Throughout central North America, anthropogenic changes in hydrologic flow networks, upland cover types, lake depths, and other factors are extensive and have been thoroughly integrated in shallow lake watersheds during the past 100 years [26], [28], [78]. Attempts to identify factors causing further changes in response to present landscape patterns may be limited when studies are based on already-impacted lakes. We think this problem presents serious challenges for ecological lake studies and may mask important causal relationships.

We found consistent negative relationships between invertebrate taxa and abundance and presence of fish communities without regard to feeding guild status. In addition, our data seem to support the hypothesis that fish presence in shallow lakes had more widespread influence on abundance of aquatic invertebrates than did densities of planktivorous or benthivorous fishes. This result is consistent with comparisons of lakes with and without fish [44], [45], [46], [80] but seems contrary to descriptions of density-dependent relationships shown between abundance of fish and zooplankton in pelagic zones of lakes ([48], [81], [82] (but not universally [83]). Despite extensive study of trophic interactions in lakes, we suggest there is still a need for replicated studies of fish-invertebrate relationships along gradients of nutrient availability, fish community compositions and densities, and plant community complexity, especially for shallow lakes.

We expected, but did not observe, a contrasting positive relationship between piscivore biomass and abundance of large cladocera. Also, post-hoc analyses indicated that residuals from our large cladocera models were poorly predicted by mass of piscivores, despite other regional data showing opposite patterns and transmission of food web effects to the zooplankton level [19], [47]. We suspect the lack of a strong relationship between large cladocera and piscivores in our models results from 3 things. First, benthivore CPUE is often positively associated with piscivores in shallow lakes [47], so it may be difficult to separate influences of these fish guilds. Surface-water connectivity and depth control fish distribution in shallow lakes [84]. When connectivity is sufficient to allow colonization by piscivores, benthivores probably use the same travel corridors and also become established; YOY benthivores probably forage on zooplankton [85]. Second, piscivore recruitment seems low in these lakes; stronger recruitment is probably necessary for control of soft-rayed planktivores. Third, piscivores do not limit populations of spiny-rayed planktivores (such as yellow perch and bluegill *Lepomis macrochirus*) in shallow Minnesota lakes [47], and these fish comprised a large share of the planktivore guild here, occurring in 36% and 14% of our sites [84].

We also detected positive associations between submerged plant mass and numbers of Amphipoda, Diptera, and Corixidae. Relatively high macroinvertebrate and zooplankton densities are known to occur in lake littoral regions [14], [54] or in shallow lakes with high submerged plant density [49]. Submerged plants are usually considered to be the most influential macrophyte component in shallow lakes [23]. Submergent plants have complex influences on chemical and physical environments and mediate strength of food web interactions by providing periphyton food resources, and day-time refugia from fish predation for macroinvertebrates and large-bodied zooplankton, at least in north-temperate shallow lakes [23], [86]. Influences of floating leaf macrophytes may also be important, but are so unique that lakes dominated by floating leaf forms have been suggested as examples of a third alternative stable regime [87]. No lakes in the present study supported large areas of floating leaf macrophytes and in a vast majority of our sites emergent plants (*Typha spp*., *Scirpus spp.*, *Phragmites australis*, and many others) were restricted to narrow shallow areas along lake margins. It is important to note that, because we were interested in broad structuring influences of submerged plant communities in study lakes, our analyses focused on lake-wide comparisons and only on combined mass of all submergent vascular species (and *Chara spp.*). Submerged macrophytes in shallow lakes contribute to regime dynamics and hysteresis [23], [52]. It is likely that even strong associations between fish and invertebrates in our study were at least partially dampened by submerged macrophytes which were abundant in many of our study lakes.

Changing abundance of aquatic invertebrates is important for conservation efforts because these animals play key roles in maintaining natural patterns of energy transfer, food chain support, and regime stability in shallow lakes. For example, suppression of large-bodied zooplankton and macroinvertebrates by planktivorous and benthivorous fishes favors higher turbidity, lower sub-surface light penetration, increases in inedible phytoplankton, and increased nutrient cycling, often facilitating shifts to turbid regimes in shallow lakes [23], [52], [62]. Zooplankton communities are often critical to maintenance of water and habitat quality in shallow lakes and lake rehabilitation often depends upon robust increases in densities of large-bodied cladocera [17], [23], [53].

Freshwater ecosystems are interdependent and fundamentally linked to terrestrial habitats [88], [89]. Development of more effective shallow lake management approaches will require understanding of multiple synergistic influences operating at lake- and watershed-scales. Field studies are always constrained by the breadth of variables they consider. This limits interpretation of ecological significance of causal relationships from variables studied singularly yet functioning synergistically. For example previous studies of shallow Minnesota lakes [49], [51] described relationships between invertebrate community pattern and 5 or 6 site-level variables for 20 lakes, but did not address potential influences of land-use in lake watersheds. Earlier work showed cumulative effects of numerous regional watershed characteristics (although not only for shallow lakes), but did not incorporate potential influences of in-lake fish communities, now known to have major structuring roles [4].

Future management strategies for shallow lakes should strive for conservation of ecosystem integrity through preservation of species and ecological processes. Approaches should emphasize not only conservation of traditional wildlife populations, but must incorporate concern for other system elements such as aquatic invertebrates because these communities are critical for sustaining ecosystem functions [1], [8]. Such a broad ecosystem approach has recently been proposed as a basis for future conservation of wetlands in North America [1]. We concur that this is needed to meet society’s expectations, to preserve natural lake functions, and to help achieve traditional conservation goals such as preservation of wildlife habitat. Identifying and establishing the magnitude of factors regulating aquatic invertebrate populations in shallow lakes seems like a necessary step toward integrating these organisms into habitat conservation approaches.
